# Surface functionalized nanoparticles of NVP an improved strategy to tackle deadly HIV infection

**DOI:** 10.1186/1471-2334-14-S3-E36

**Published:** 2014-05-27

**Authors:** Bhagyashree R Dalvi, Shilpa M Velhal, Atmaram B Bandivadekar, Absar Ahmad, Padma V Devarajan

**Affiliations:** 1 Department of Pharmaceutical Sciences and Technology, Institute of Chemical Technology, Mumbai, India; 2 National Institute for Research in Reproductive Health, Mumbai, India; 3 Biochemical Sciences, National Chemical Laboratory (CSIR), Pune, India

## Background

The therapeutic efficacy of nevirapine (NVP) is hampered by its poor solubility; poor ability to target infected cells and inherent toxicity. The objective of the present study was surface functionalization with a new targeting ligand to enable enhanced efficacy.

## Methods

Surface functionalization of GMS nVP auNPs was studied by fluroscence spectrophotometry. Nanoformulations and NVP were evaluated for cytotoxicity at 1, 2 and 24 hours and uptake study at 0.5, 1 and 2 hours. *In vitro* anti-HIV activity was evaluated in TZM bl cell line against HeLa/LaV virus.

## Results

GMS nVP auNPs with > 75% entrapment efficiency & < 300nm particle size were prepared and surface modified nanoparticles showed >85% binding to the ligand. In cytotoxicity study nanoformulations showed higher cell viability at all time points as compared to NVP. Cell uptake study revealed higher uptake of nanoparticles as compared to NVP only. In vitro anti HIV assay of surface modified revealed 10 fold increased activity as compared to NVP.

## Conclusion

Nanosize and surface functionalization both play an important role in enhancing anti HIV potential of GMS nVP auNPs.

